# Item response theory analysis of the Dysfunctional Beliefs and Attitudes about Sleep-16 (DBAS-16) scale in a university student sample

**DOI:** 10.1371/journal.pone.0281364

**Published:** 2023-02-02

**Authors:** Louise I. R. Castillo, Thomas Hadjistavropoulos, L. Odell Tan, Ying C. MacNab

**Affiliations:** 1 Department of Psychology and Centre on Aging and Health, University of Regina, Regina, Canada; 2 Department of Psychiatry and Behavioural Neurosciences, McMaster University, Hamilton, Canada; 3 School of Population and Public Health, University of British Columbia, Vancouver, Canada; Sapienza University of Rome: Universita degli Studi di Roma La Sapienza, ITALY

## Abstract

Unhelpful beliefs about sleep have been shown to exacerbate distress associated with sleep-related difficulties. University students are particularly vulnerable to experiencing sleep-related problems. The Dysfunctional Beliefs and Attitudes about Sleep-16 (DBAS-16) scale is a widely used instrument that assesses for sleep-disruptive cognitions. Although psychometric support for the DBAS-16 is available, Item Response Theory (IRT) analysis is needed to examine its properties at the item level. Psychometric investigation in non-clinical samples can help identify people who may be at risk for developing sleep problems. We examined the DBAS-16 using IRT on a sample of 759 university students. Our results identified items and subscales that adequately/inadequately differentiated between students who held unhelpful beliefs about sleep and those who did not. The DBAS-16 is a valuable instrument to assess unhelpful beliefs about sleep. We outline recommendations to improve the discriminatory ability of the instrument. Future investigations should establish cross-validation with a clinical sample.

## Introduction

Sleep-related difficulties affect a large proportion of the population and are associated with impairments in interpersonal, physical, and social functioning [1–4]. University students, in particular, are vulnerable to experiencing sleep problems [5]. In a survey of university students in the nited States, 43% reported difficulties staying asleep (e.g., waking up more than once in the middle of the night) [6]. Similar results have been obtained in other countries [7]. This is concerning given that sleep difficulties among university students are related to increased mental health problems [8–10]. In addition, reduced duration and quality of sleep among university students have been shown to negatively impact daily activities and academic performance [11–13]. Even among students with generally healthy sleeping habits, reduced sleep quality is associated with poorer mental health outcomes [14]. Given the high prevalence of sleep problems among university students, sleep-related measures have been developed for and tested in this population [15, 16]. In fact, use of a measure of attitudes about sleep should not be restricted to clinical populations but may be useful for the identification of people who may be at risk for developing insomnia later on. As the cognitive model of insomnia posits, unhelpful beliefs about sleep exacerbate intrusive worries about lack of sleep which, in turn, trigger physical arousal and interferes with ability to fall asleep [17]. As such, psychometric analyses of scales concerning dysfunctional attitudes about sleep are important to validate in student and other non-clinical populations so that those at risk for developing future sleep problems can be identified and offered appropriate preventative interventions and guidance.

One of the widely used instrument used to examine problems and difficulties related to sleep is the Dysfunctional Beliefs and Attitudes about Sleep (DBAS) scale. Morin [18] developed the 30-item scale to assess misconceptions about the causes of insomnia, misattributions of the consequences of insomnia, and unrealistic sleep expectations. Morin et al. [19] later presented an abbreviated 16-item (DBAS-16) version of the original 30-item scale to ease the administration and improve the utility of the scale. Since their development, the DBAS scales have been widely used and tested in clinical and nonclinical samples [20–23]. Moreover, the original and abbreviated scales have been translated into multiple languages [24–27]. The DBAS-16 particularly targets the following sleep-disruptive cognitions: expectations about sleep requirements, attributions of the causes and appraisals of the consequences of insomnia, and issues of worry and helplessness about sleep [19]. The DBAS-16 has been used among university students where elevated levels of unhelpful beliefs and attitudes about sleep were identified [28]. Although there are no cut-off scores in the current scoring guidelines of the measure [19], previous research has identified that, using a 0–10 scale, total scores of 4 or higher may indicate unhelpful beliefs and expectations about sleep [21, 29, 30]. The DBAS-16 has also been shown to be valid in discriminating between patients with insomnia and controls [21, 31]. It is also sensitive to the effects of treatment [32–34].

The psychometric properties of the DBAS scales have been studied [18, 19, 24, 27, 35] and concerns about several DBAS-16 items have been noted. For example, Items 10 (“I can’t ever predict whether I’ll have a good or poor night’s sleep”), 11 (“I have little ability to manage the negative consequences of disturbed sleep”), and 16 (“I avoid or cancel obligations (social, family) after a poor night’s sleep”) have been shown to have low factor loadings under the worry/helplessness about insomnia (Items 10 and 11) and perceived consequences of insomnia (Item 16) subscales but were retained due to their clinical relevance [19, 25]. Moreover, the 2-item sleep expectation subscale, which includes Item 1 (“I need 8 hours of sleep to feel refreshed and function well during the day) and 2 (“When I don’t get the proper amount of sleep on a given night, I need to catch up the next day by napping or the next night by sleeping longer”), has been shown to have significantly low factor loadings but was retained due to its utility in treatment planning for individuals with insomnia [19, 25, 36]. Furthermore, Carney and Edinger [37] highlighted concerns about multiple items in the DBAS scales not adequately differentiating between good and poor sleepers.

Commensurate with the need to evaluate treatments that address maladaptive cognitions in individuals with sleep related difficulties is the need to develop and evaluate reliable and theoretically sound instruments capable of assessing treatment effectiveness. Considering that the DBAS-16 developers called for an item response theory (IRT) analysis of the scale [19] as well as the item specific concerns outlined in the literature, we set out to further investigate the psychometric properties of the DBAS-16 in a student sample vulnerable to experiencing sleep problems. Moreover, the DBAS-16 could help with the early identification of individuals with sleep problems and, as such, would be important to study in non-clinical samples. IRT places emphasis on the properties of each item of the scale as opposed to a test level focus (e.g., total score) which is the basis of classical test theory [38]. We conducted an IRT analysis to examine the probability of item endorsement as a function of degree of unhelpful beliefs and attitudes about sleep by examining each subscale. In particular, the objective of our investigation was to identify the subscales and items that best discriminate between university students with low vs. high maladaptive beliefs about sleep.

## Methods

All procedures performed in studies involving human participants were in accordance with the ethical standards of the University of Regina Research Ethics Board (# 2015–093). Written informed consent was obtained from all individual participants included in the study using an electronic online form.

### Participants

Data from this investigation were collected as part of a larger study of 765 university student participants [15]. In the context of the larger study supporting the validity of the DBAS-16 in a university sample, its scores were positively correlated with higher levels of daytime sleep worry (Insomnia Daytime Worry Scale [39]; M = 14.83, SD = 11.34), neuroticism (Neuroticism scale of the Big Five Inventory [40]; M = 3.18, SD = 0.75), depressive symptoms (Center for Epidemiologic Studies Depression Scale–10 [41]; M = 9.94 SD = 5.30), worse sleep quality (Pittsburgh Sleep Quality Index [42]; M = 7.58, SD = 3.14), and perception of sleep problems (Insomnia Severity Index [43]; M = 9.79, SD = 5.52) [15].

Participants were recruited using a student-wide electronic mailing list of our institution and were offered an opportunity to be entered in a draw for one of four $50 gift cards to either a department store or a restaurant. There were no other specific inclusion or exclusion criteria. Potential participants were provided with a link to the study through Qualtrics Survey Software that led to an informed consent form, demographic information, and of the aforementioned questionnaires, including the DBAS-16. Six participants with incomplete DBAS-16 responses were excluded from the final sample. Furthermore, the proportion of missing data for each item of the DBAS-16 was less than 5–10% (i.e., less than 0.3% in our sample) which is consistent with published guidelines for deletion of cases (e.g., [44, 45]). As such, a complete case analysis was conducted. The omitted participants represented 0.8% of the overall sample. After the deletion of the six incomplete participant data sets, a total of 759 participants were included in this study. The mean age of our sample was 23.39 (SD = 6.89), and 77.3% were female. Participants had completed an average of 14.83 years (SD = 3.84) of formal education.

### Measures

#### Dysfunctional Beliefs and Attitudes about Sleep-16 (DBAS-16)

The Dysfunctional Beliefs and Attitudes About Sleep–16 (DBAS-16) [19] is a 16 item self-report questionnaire designed to measure various maladaptive sleep-related beliefs and attitudes an individual may hold. In this study, we used a Likert scale with scores ranging from 1 (Strongly disagree) to 10 (Strongly agree). This scale consists of four subscales which corresponds to the four-factor model that has been identified for the scale: 1) sleep expectations; 2) worry/helplessness about insomnia; 3) perceived consequences of insomnia and 4) medications. Scoring involves the following: 1) an average overall score (i.e., obtained by adding all the scores for all the items and dividing by 16); and 2) an average subscale score (i.e., obtained by summing the score of each subscale and dividing by the number of items per subscale) [19]. The degree of unhelpful beliefs is reflected in the strength of endorsement for each item (i.e., a higher rating indicates stronger agreement to an item). The DBAS-16 has shown adequate internal consistency in clinical samples (a = 0.77; [19]) and among university students (a = 0.82; [29]). Validity in discriminating between people with and without insomnia and has also been demonstrated [21, 31]. For our sample, the overall scale illustrated strong internal consistency (Cronbach’s alpha = 0.861) and internal consistencies of as the subscales were as follows: 0.559 (expectations); 0.792 (worry/helplessness); 0.781 (consequences); and 0.523 (medications)

### Analysis

#### Confirmatory factor analysis

To examine model fit and in accordance with the factors previously identified for this scale, confirmatory factor analyses (CFA) were conducted using AMOS (SPSS 26.0). Previous research has supported a four-factor structure model fit of this scale [19].

#### Item response theory analysis

Item Response Theory (IRT) is grounded on the concept that estimates of an individual’s latent ability or theta (θ) are dependent on how one responds to the item and the properties (e.g., difficulty of item) inherent to the items in the instrument [46]. IRT deviates from Classical Test Theory (CTT) in various ways, namely, CTT uses the true score and the error to directly predict an individual’s total score with the assumption that item properties are equivalent across an instrument and that item properties are not linked to behavior [46]. In contrast, IRT identifies a person’s trait level based on individual response patterns and locates both the individual and the scale item in the same continuum of an underlying construct (e.g., trait level) [46]. The ability to examine how item properties influence trait measurement has led to the varied use of IRT analysis in the development and refinement of various measures [15, 47].

The graded response model (GRM) [48] was formulated for the analysis of items consisting of two or more ordered categorical responses (e.g., a Likert Scale), such as the DBAS-16 items considered in this paper. In a GRM, each item is characterized by a slope parameter and a set of between category threshold parameters b_1_, …, b_C-1_, where C is the number of item response categories (e.g., for the DBAS-16 items, C = 10). The slope parameter reflects the strength of the relationship of the item to the latent variable. Each of the threshold parameter quantifies the trait level necessary to have 0.50 probability of choosing a response above a given score (e.g., the level of unhelpful belief and attitude about sleep needed to have an equal probability of choosing strongly disagree [1] or higher) [49]. In the present study, IRTPRO (version 4.20) was used to conduct the analysis using the GRM with 9 threshold parameters for each of the items.

## Results

Table 1 outlines the descriptive statistics of the overall, item, and subscale scores for our sample.

**Table 1:** Item, subscale, and overall scores for the DBAS-16.

	Mean	Standard Deviation
**Total Score**	4.74	1.47
**Expectations Subscale**	6.76	2.26
Item 1	7.17	2.63
Item 2	6.34	2.80
**Worry/Helplessness Subscale**	4.25	1.86
Item 3	4.73	3.00
Item 4	3.62	2.59
Item 8	3.68	2.55
Item 10	5.63	2.88
Item 11	4.57	2.44
Item 14	3.25	2.43
**Consequences Subscale**	5.06	1.81
Item 5	6.37	2.53
Item 7	5.13	2.51
Item 9	4.42	2.47
Item 12	6.00	2.34
Item 16	3.40	2.58
**Medication Subscale**	3.82	1.79
Item 6	4.02	3.04
Item 13	4.92	2.21
Item 15	2.53	2.16

**Note**. DBAS-16 = Dysfunctional Beliefs and Attitudes about Sleep-16 Scale

### Confirmatory factor analysis

Initial results of the four-factor CFA revealed poor model fit: CFI = .876, RMSEA = .080, χ2(98) = 573.75, *p* < .001. As such, items with low factor loadings (i.e., less than 0.55) were dropped to improve model fit and gain model identification. After omitting Items 10 (0.43), Item 16 (0.52), and Item 13 (0.32), the model fit for the four-factor structure improved significantly: CFI = 0.918, RMSEA = 0.078, SRMR = 0.054l, χ2(58) = 326.44, *p* < .001. The final standardized factor loadings ranged from 0.56 (Item 3) to 0.80 (Item 15). The progression of item removal and factor loadings are outlined in Table 2. The final items were included in the IRT analysis.

**Table 2:** Four-factor confirmatory factor analysis of the DBAS-16.

Subscale and item number	Subscale loading	
	Original	Final
**Expectations**		
Item 1	0.58	0.58
Item 2	0.67	0.67
**Worry/Helplessness**		
Item 3	0.62	0.56
Item 4	0.75	0.71
Item 8	0.66	0.68
Item 10 ^a^	0.43	
Item 11	0.57	0.57
Item 14	0.76	0.75
**Consequences**		
Item 5	0.74	0.75
Item 7	0.61	0.62
Item 9	0.77	0.78
Item 12	0.60	0.61
Item 16 ^a^	0.52	
**Medication**		
Item 6	0.58	0.58
Item 13 ^a^	0.36	
Item 15	0.75	0.80

Note.

^a^ = Item removed to improve subscale fit.

An additional correlation (0.31) was added between the errors of Items 3 and 4. DBAS-16 = Dysfunctional Beliefs and Attitudes about Sleep-16 Scale.

### Item response theory analysis

The perceived consequences of insomnia subscale included items 5, 7, 9, and 12. The model fully converged at a statistically significant level: AIC = 12629.33, BIC = 12814.62, M_2_(482) = 673.92, RMSEA = 0.02, p < 0.01. The worry/helplessness about insomnia subscale included items 3, 4, 8, 11, and 14. The model fully converged at a statistically significant level: AIC = 16790.96, BIC = 15022.56, M_2_(805) = 1099.14, RMSEA = 0.02, p < 0.01. The expectations about sleep subscale included items 1 and 2. The model fully converged at: AIC = 6436.78, BIC = 6529.42, M_2_(79) = 97.81, RMSEA = 0.02, p = 0.0743. The medication subscale for this analysis included items 6 and 15. The model fit for the medication subscale fully converged at: AIC = 5425.73, BIC = 5518.37, M_2_(79) = 77.73, RMSEA = 0.00, p = 0.5201.

The IRT analyses were conducted according to the final four-factor structure. For brevity, Table 3 illustrates the item specific estimates (mean and standard errors) of the slope parameter (a) and two of the nine category threshold parameters (i.e., item discrimination parameters b_1_ and b_9_). Items of higher slope parameters contribute more item information to the scale. The slope parameter estimates ranged from 1.29 (Item 2) to 3.42 (Item 15). The threshold parameter estimate reflects item difficulty or the extent to which a respondent with a given latent trait has an equal probability of endorsing an item [46]. The b_1_ parameter estimate indicates the average latent ability threshold necessary for rating items as Strongly disagree or higher. Across all the subscales, this ranged from -2.64 (Item 12) to 0.02 (Item 15). Similarly, the b_9_ parameter estimate indicates the average latent ability threshold necessary for rating items as Strongly agree or lower response options. Estimates ranged from 0.80 (Item 1) to 3.18 (Item 11). For Item 15, the standard error for the b_9_ parameter was high (Mean = 2.33, standard error = 6.29). This is likely due to low frequencies of response score 9 (0.5%) and 10 (2.0%) in our data; these response scores have the lowest response frequencies among the 16 items. Moreover, 50.5% of respondents scored Item 15 as a 1 (strongly disagree) and the majority of respondents disagreed.

**Table 3:** Summary of item parameters estimates.

Subscale and item number	Item parameters		
	*a*	*b1*	*b9*
**Expectations**			
Item 1	2.01 (0.54)	-2.43 (0.32)	0.80 (0.11)
Item 2	1.29 (0.23)	-2.50 (0.34)	1.57 (0.20)
**Worry/Helplessness**			
Item 3	1.82 (0.13)	-1.01 (0.08)	1.87 (0.12)
Item 4	2.87 (0.24)	-0.62 (0.06)	2.17 (0.13)
Item 8	1.44 (0.11)	-0.96 (0.09)	2.75 (0.20)
Item 11	1.16 (0.09)	-2.05 (0.17)	3.18 (0.26)
Item 14	2.50 (0.20)	-0.44 (0.06)	2.45 (0.15)
**Consequences**			
Item 5	2.07 (0.16)	-2.33 (0.15)	1.44 (0.09)
Item 7	1.63 (0.12)	-1.94 (0.14)	2.58 (0.18)
Item 9	2.11 (0.16)	-1.37 (0.08)	2.44 (0.15)
Item 12	1.74 (0.13)	-2.64 (0.18)	2.00 (0.13)
**Medication**			
Item 6	1.50 (0.19)	-0.62 (0.11)	2.32 (0.25)
Item 15	3.42 (0.21)	0.02 (0.05)	2.33 (6.29)

**Note**. *a* (item discrimination estimate) = indicates the strength of the item to the latent variable; *b1* (item difficulty estimate) = indicates the average latent ability necessary to have a 0.50 probability of choosing 1 (strongly disagree) or higher; *b9* = indicates the average latent ability necessary to have a 0.50 probability of choosing 10 (strongly agree) or a lower score. Values in parenthesis are item parameter standard error estimates.

Furthermore, IRTPRO calculates an item information function for each item. Item information function reflects the level of precision or information that each item provides to the overall scale and locates the precision across the underlying trait continuum [49]. As such, an item that adequately discriminates among individuals along the trait continuum is expected to provide the highest degree of information for the level of latent variable (i.e., those with unhelpful beliefs and attitudes about sleep) that the item aims to examine (e.g., between the range θ = 1.00 to θ = 3.00). Item information functions can therefore be used to identify items that are most helpful to the overall scale [49]. The item characteristic curve and item information function of each item were scoped to examine curve patterns and parameter estimates.

A summary of item information estimates is outlined in Table 4. Item 14 provided the most information (1.01) among all the items for individuals on the extreme end of the spectrum (θ = 3.00) (i.e., high levels of worry about sleep). For the expectations subscale, Item 1 provided the most information about respondents with average levels of expectations about sleep. Item 4 in the worry/helplessness subscale provided the most information for individuals that excessively worry about sleep (i.e., θ = 0.00 and θ = 2.00). For the consequences subscale, Item 9 provided the most information among those with high level of unhelpful beliefs about the negative consequences of insomnia (i.e., θ = 0.00 and θ = 2.00). For the medication subscale, Item 15 provided significant information about respondents who heavily attribute relief from sleep-related distress to medications alone.

**Table 4:** Summary of item information estimates.

Subscale and item number	Latent ability (theta; θ)						
	-3.00	-2.00	-1.00	0.00	1.00	2.00	3.00
**Expectations**							
Item 1	0.76	1.29	1.32	1.30	1.05	0.31	0.05
Item 2	0.40	0.53	0.55	0.55	0.53	0.41	0.20
**Worry/Helplessness**							
Item 3	0.08	0.41	0.96	1.08	1.08	0.91	0.34
Item 4	0.01	0.15	1.57	2.64	2.68	2.53	0.63
Item 8	0.10	0.32	0.59	0.68	0.69	0.67	0.55
Item 11	0.26	0.39	0.43	0.44	0.44	0.43	0.40
Item 14	0.01	0.12	1.00	1.98	2.03	1.99	1.01
**Consequences**							
Item 5	0.69	1.33	1.39	1.38	1.35	0.79	0.16
Item 7	0.34	0.74	0.86	0.86	0.85	0.83	0.62
Item 9	0.14	0.75	1.38	1.44	1.43	1.40	0.82
Item 12	0.72	0.96	0.99	0.98	0.97	0.87	0.39
**Medication**							
Item 6	0.06	0.22	0.55	0.72	0.74	0.70	0.45
Item 15	0.00	0.01	0.34	3.24	3.75	3.72	0.99

**Note**. Item information estimate indicates the degree of information or level of precision an item adds to the overall scale across the underlying latent variable (theta; θ).

**Fig 1:**
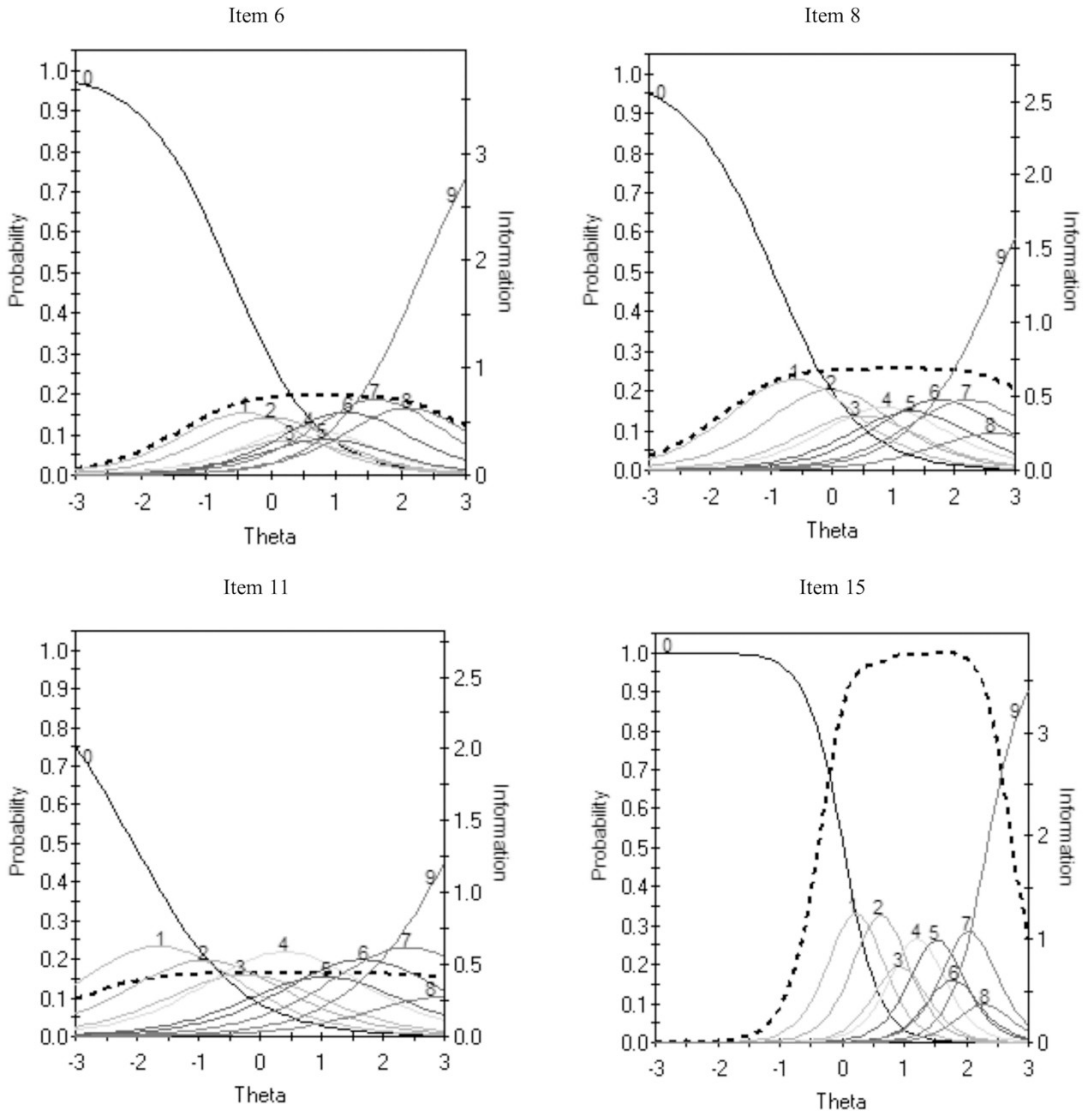
Item characteristic curves for dysfunctional Beliefs and Attitudes about Sleep (DBAS-16) scale items 6, 8, 11 and 15. The left y-axis represents the probability of choosing a response category and the right y-axis represent the degree of information provided across the underlying trait continuum. The peak of each response function represents the maximum probability of choosing that response category given a theta level (i.e., underlying trait). Item information function is indicated with a dashed line. IRTPRO (Version 4.2) by default shows response alternatives (strongly disagree [1] to strongly agree [10]) as 0 to 9.

Of note, Items 6, 8, and 11 had characteristically flat and unique curves in comparison to all the other items. Fig 1 illustrates this by contrasting their item characteristic curves with that of the Item 15. Item 15 had the highest item discriminate estimate (i.e., the best item to discriminate from high vs low dysfunctional beliefs) and provided the most information across individuals with unhelpful beliefs (i.e., 0.99–3.75).

**Fig 2:**
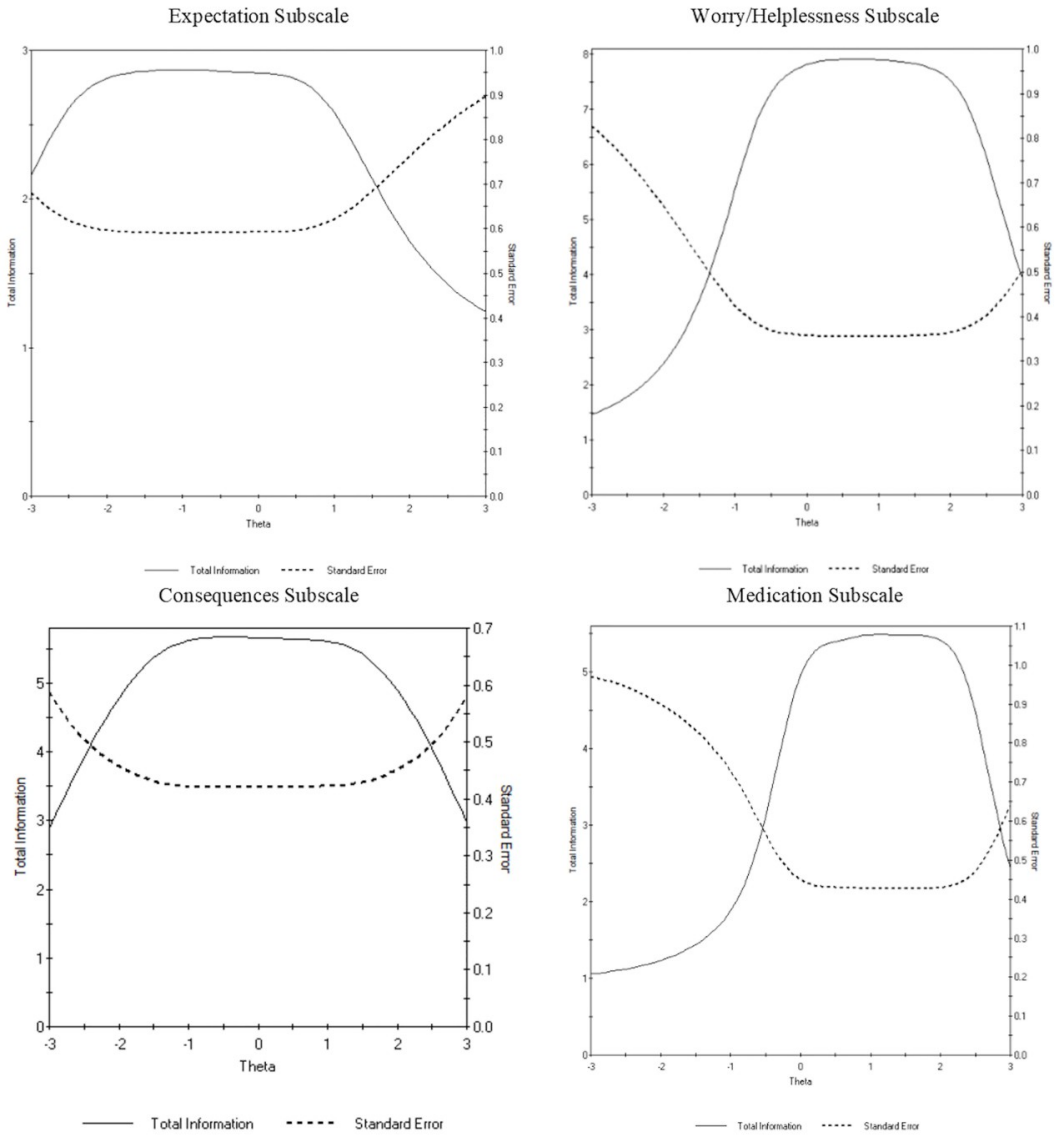
Test information function of the expectations, worry/helplessness, consequences, and medication subscales. The peak of the total information curve represents the theta level (i.e., degree of unhelpful beliefs and attitudes about sleep) at which the Dysfunctional Beliefs and Attitudes about Sleep (DBAS-16) Scale most accurately predicts degree of dysfunctional beliefs and attitudes about sleep (i.e., worry, expectations, attributions, consequences). The peak of the standard error curve represents the theta level at which the DBAS-16 has the most error in predicting the degree of dysfunctional beliefs and attitudes about sleep.

IRTPRO also calculates test information curves for each subscale. The item information estimates for each item are summed together to create a total information function. As with the item information estimate, the test information curves can provide information about the scale as a function of the location on the trait continuum [49]. Fig 2 outlines the test information curves for each subscale. The items in the expectation subscale exhibited low test information estimates across the full range of the latent trait (θ = 3.00 and θ = 3.00). Similarly, the medication subscale had low test information estimate in most of the negative range of the latent trait (θ = 3.00 and θ = -0.50). Items in the consequences subscale provided the most information about respondents with latent levels between approximately θ = -1.50 and θ = 1.50. The worry/helplessness subscale provided the most information about respondents with latent levels approximately between θ = -0.50 and θ = 2.00 and the highest peak of total information estimate (i.e., the information estimate ranged from 1.5 to 8.0) among all the subscales.

## Discussion

To the best of our knowledge, this study represents the first IRT analysis of the DBAS-16 scale. According to the results and in accordance with the objective of the study, we identified items and subscales that adequately discriminated between those with low and high levels of unhelpful beliefs and attitudes about sleep in a university student sample. The items identified may elucidate understanding on the sleep-related cognitions germane to university students. Items 10 (“I can’t ever predict whether I’ll have a good or poor night’s sleep”), 13 (“I believe insomnia is essentially the result of a chemical imbalance”) and 16 (“I avoid or cancel obligations [social, family] after a poor night’s sleep”) were omitted from the IRT analysis due to low factor loadings. Low factor loadings for Items 10 and 16 are consistent with previous research in non-clinical samples [25]. A proposed explanation for Item 16 has been the change in appraisal for the potential consequences of canceling obligations due to insufficient sleep as a result of alternate forms of communications (e.g., social media) [25]. That is, university students may not view avoiding or canceling social obligations as a consequence of insufficient sleep because they have access to technology as means for continuing communication.

The item characteristic curves for Items 6 (“To be alert and function well during the day, I believe I would be better off taking a sleeping pill rather than having a poor night’s sleep”), 8 (“When I sleep poorly one night, I know it will disturb my sleep schedule for the whole week”), and 11 (“I have little ability to manage the negative consequences of disturbed sleep”) had characteristically flat curves in comparison to the rest of the items, particularly for average to moderately high response levels. The lack of well-defined or unique peaks across each response category (i.e., score ratings 2 to 9) suggest that these response options are not being utilized as intended and therefore lack predictive value and discriminatory power. In addition, there is approximately the same probability of choosing response options 2 to 9 among these items, suggesting that the items do not adequately discriminate among students with varying degree of unhelpful beliefs about sleep. This is also consistent with the item information curves of Items 6, 8, and 11 which indicated that the items provided little information with low precision and low score accuracy to the overall scale. These findings may be characteristic of our university student sample. For instance, a survey found that although 43% of students experienced sleep difficulties, only 10% of their sample reported using other aids (e.g., medication) to improve their sleep [6]. On the other hand, other research has shown more frequent use of medication among students to alleviate sleep problems [5]. The lack of defined endorsement for certain response options may be due to the varied use of sleeping aids among university students. For Item 8, variable sleep schedules and patterns have been documented among university students [5, 50]; as such, students may not hold strong beliefs about the effects of sleep variability on the quality of their sleep. Nonetheless, future investigations could explore ways of improving the instrument by refining these items so that they can better discriminate between people who hold unhelpful attitudes about sleep and people who do not. Item 11 had the lowest discriminatory value among all the items indicating it does not appear to adequately differentiate among students with varying levels of worry about sleep. Moreover, further research is necessary to ascertain a 4-factor construct and to conduct a multi-dimensional IRT for factor structure assessment.

### Expectations and medication subscales

Of the four subscales, low test information (e.g., estimated total information values less than 3) were observed in the expectations subscale for the full range of the latent trait and the medication subscale in most of the negative range of the latent trait (e.g., individuals who do not attribute insomnia to a chemical imbalance or medication). Overall, our statistical findings are consistent with those presented in Morin et al. [19] where the 16 DBAS items were subjected to a CFA. Morin et al. [19] reported notably lower CFA coefficient (standard estimate) for the expectations (B = 0.50) and medication (B = 0.43) subscales. Despite identifying low item-total correlations for a few items within the medication and expectations subscales in the development of the DBAS-16, the items were retained due to their content validity and clinical relevance among a subgroup of individuals with insomnia [19]. As such, a possible explanation for the low test information estimates for the expectations and medication subscales could be that our nonclinical sample comprised of individuals experiencing varying levels of sleep difficulties, with a small subset potentially meeting criteria for insomnia. This finding also underscores the notion that the expectations and medication subscales may be more appropriate in identifying the degree of specific unhelpful beliefs among individuals with known insomnia to aid in treatment planning (e.g., cognitive therapy for insomnia) rather than identifying unhelpful beliefs about sleep among individuals who may be vulnerable to developing sleep related issues. Since the DBAS-16 was developed to stimulate greater use of the DBAS among the sleep community [18], understanding its utility in various settings and with non-clinical samples is of importance.

Examining the items in both subscales also offers opportunities for item refinement. In our analysis, Item 13 (“I believe insomnia is essentially the result of a chemical imbalance”) was removed from the medication subscale IRT analysis due to low factor loading (B = 0.36), which seems to have contributed to the failed convergence of the original IRT fit. This is consistent with the CFA performed by Morin et al. [19] where the authors also reported low factor loading for Item 13 (B = 0.06). As discussed above, Item 6 (“To be alert and function well during the day, I believe I would be better off taking a sleeping pill rather than having a poor night’s sleep”), in the medication subscale also led characteristically flat item characteristic curve in comparison to the rest of the items, suggesting that the item lacks predictive value and discriminatory power. Item 15 (“Medication is probably the only solution to sleeplessness”) had the highest discriminatory value for the underlying construct indicating that this item adequately discriminates among individuals who believe that medication is the only solution to sleeplessness from those who do not. This finding is consistent with the item difficulty estimate; in order to have an equal probability (i.e., 0.50) of rating Item 15 as either 1 or higher (i.e., Strongly disagree or higher), an individual would have to hold some unhelpful attributions of causes of insomnia (b1 = 0.20). This suggests that this item is “difficult”, and any degree of agreement reflects unhelpful causal attributions of insomnia (i.e., believing that medication is the solution to sleeplessness). However, our data also led to a high standard error for the b_9_ estimate of Item 15, which is likely due to low frequencies of high response scores for this item. As such, caution must be taken when interpreting the discriminatory ability of Item 15. The high standard error for Item 15 and low frequencies for high response options also corroborates the idea that our sample may have consisted of individuals experiencing varying levels of sleep difficulties, with a subset potentially meeting criteria for insomnia.

The items within the expectations subscale (Item1 [“I need 8 hours of sleep to feel refreshed and function well during the day] and Item 2 [“When I don’t get the proper amount of sleep on a given night, I need to catch up the next day by napping or the next night by sleeping longer”]) provided greater information about individuals without unrealistic expectations about sleep. The items within this subscale have been retained because they represent maladaptive cognitions expressed by subgroups of people with insomnia and could inform treatment [19]. Our results are consistent with previous research [19, 25, 36] demonstrating low factor loadings and extend previous findings by highlighting item specific issues within this subscale using an IRT analysis.

A possible explanation for these item specific issues may be the semantics or the content of each item. Modifying the presentation of each item or creating more stringent respondent criteria could aid in improving its discriminatory ability to assess broader groups of people experiencing sleep difficulties. For example, the idea of needing to sleep for eight hours may be a common expectation and as such, a strong endorsement of Item 1 may not indicate unhelpful attitudes. It should be noted that in this sample, over 68% of the participants rated this item as 7/10 or greater. Modifying the item to “I should **always** get 8 hours of sleep” may be useful. Similarly, the idea of needing to compensate as a result of not attaining the “proper amount of sleep” (Item 2) may be unclear. Perhaps considering changing the item to “I need to get the proper amount of sleep on a given night or else I will need to catch up the next day by napping or the next night by sleeping longer” could be helpful.

### Consequences and worry/helplessness subscales

Our statistical findings are consistent with those presented by Morin and colleagues [18] who reported high CFA coefficient for the consequences (0.94) and worry/helplessness (0.88) subscales. Furthermore, total item information curves demonstrated that the items within the worry/helplessness subscale provided the most information and highest level of precision about individuals that excessively worry about sleep, followed by the consequences subscale. This suggest that, perhaps, the items within the worry/helplessness and consequences subscales best represent the items needed to measure unhelpful beliefs and attitudes about sleep among university students. This finding will need to be re-evaluated in a clinical sample. However, we also identified characteristically flat curves for Items 8 and 11 in the worry/helplessness subscale which suggest opportunities for item and response category refinement. The items in the consequences and worry/helplessness subscales could be useful for screening for extreme unhelpful beliefs about sleep in individuals in the general population (e.g., student population) who may be vulnerable to developing sleep difficulties. Our results increase confidence in the effectiveness of the items within these subscales in discriminating between individuals who hold unhelpful beliefs about sleep at varying degrees.

The item specific issues identified in our analyses are consistent with problems raised in studies examining clinical and nonclinical samples [19, 25, 36]. That said, increased sleep-related distress has been documented among university students and this population has been identified as being vulnerable in the development of sleep problems [51, 52]. Given the high prevalence of sleep problems among university students, sleep-related measures have been developed for and tested in this population. In particular, catastrophic thoughts impacting sleep and the overall sleep quality of university students have been the subject of previous investigations [15, 16]. Consistent with previous research [28, 29] that identified similarly elevated levels of dysfunctional beliefs about sleep in student samples, our findings demonstrate that students are particularly vulnerable to the presence of dysfunctional beliefs about sleep given the mean scores obtained. Although the presence of unhelpful beliefs and attitudes about sleep does not directly result in insomnia, consistent with the cognitive model of insomnia [17], it may increase a student’s vulnerability to developing sleep-related disorders. This is concerning given that reduced quality of sleep among university students has been shown to negatively impact daily activities, academic performance, and mental health outcomes [11–13].

### Limitations

We recognize that our focus on university student research participants may limit the generalizability of our findings. At the same time, psychometric investigation of the DBAS-16 in a non-clinical sample with vulnerability to develop insomnia is important because it can be used as a screening tool to identify those at risk of developing sleep problems later on. Nonetheless, studies similar to ours, using clinical samples, are warranted.

The absence of information on certain sociodemographic characteristics (e.g., ethnicity, race) poses some limitations on our ability to generalize these results. As such, it would be important for future research to examine the impact of these characteristics on the psychometric properties of the DBAS-16. Small sample sizes may limit the assessment of a four-factor structure in an IRT analysis. Although we tried to mitigate this with a larger sample size of 759 participants, further research is needed to conduct a multi-dimensional IRT analysis for factor structure assessment and ascertain a four-factor construct.

## Conclusion

To the best of our knowledge, this study represents the first IRT analysis of the DBAS-16 scale. Overall, the DBAS-16 is a valuable instrument for assessing unhelpful beliefs and attitudes about sleep. In our university student sample, certain items within the DBAS-16 were identified to best discriminate between those with low and high levels of unhelpful beliefs and attitudes. However, various items were also shown to have low discriminatory value for identifying students with maladaptive beliefs. Items 6, 8, and 11 were identified to not be performing adequately, thus suggesting opportunities for item refinement and improvements to the overall instrument. In addition, we identified low factor loadings for Items 10, 13, and 16. Future research should examine the impact of omitting these items from instrument. Items within the expectations and medication subscales may be more effective at identifying unhelpful beliefs about sleep in clinical samples while the items in the consequences and worry/helplessness subscales are effective in general settings. Psychometric investigation of the DBAS-16 in non-clinical samples is important and can help identify people who may be at risk for developing sleep problems, future investigations should examine whether these results are replicated in samples of people who present for treatment for insomnia.
